# HCT116 colorectal liver metastases exacerbate muscle wasting in a mouse model for the study of colorectal cancer cachexia

**DOI:** 10.1242/dmm.043166

**Published:** 2020-01-24

**Authors:** Joshua R. Huot, Leah J. Novinger, Fabrizio Pin, Andrea Bonetto

**Affiliations:** 1 Department of Surgery, Indiana University School of Medicine, Indianapolis, IN 46202, USA; 2 Department of Otolaryngology – Head & Neck Surgery, Indiana University School of Medicine, Indianapolis, IN 46202, USA; 3 Department of Anatomy, Cell Biology and Physiology, Indiana University School of Medicine, Indianapolis, IN 46202, USA; 4 Indiana Center for Musculoskeletal Health, Indiana University School of Medicine, Indianapolis, IN 46202, USA; 5 IUPUI Center for Cachexia Research Innovation and Therapy, Indiana University School of Medicine, Indianapolis, IN 46202, USA; 6 Simon Cancer Center, Indiana University School of Medicine, Indianapolis, IN 46202, USA

**Keywords:** Colorectal cancer, Liver metastases, HCT116, Cachexia, STAT3, Skeletal muscle

## Abstract

Colorectal cancer (CRC) is often accompanied by formation of liver metastases (LM) and skeletal muscle wasting, i.e. cachexia. Despite affecting the majority of CRC patients, cachexia remains underserved, understudied and uncured. Animal models for the study of CRC-induced cachexia, in particular models containing LM, are sparse; therefore, we aimed to characterize two new models of CRC cachexia. Male NSG mice were injected subcutaneously (HCT116) or intrasplenically (mHCT116) with human HCT116 CRC tumor cells to disseminate LM, whereas experimental controls received saline (*n*=5-8/group). Tumor growth was accompanied by loss of skeletal muscle mass (HCT116: −20%; mHCT116: −31%; quadriceps muscle) and strength (HCT116: −20%; mHCT116: −27%), with worsened loss of skeletal muscle mass in mHCT116 compared with HCT116 (gastrocnemius: −19%; tibialis anterior: −22%; quadriceps: −21%). Molecular analyses revealed elevated protein ubiquitination in HCT116, whereas mHCT116 also displayed elevated Murf1 and atrogin-1 expression, along with reduced mitochondrial proteins PGC1α, OPA1, mitofusin 2 and cytochrome C. Further, elevated IL6 levels were found in the blood of mHCT116 hosts, which was associated with higher phosphorylation of STAT3 in skeletal muscle. To clarify whether STAT3 was a main player in muscle wasting in this model, HCT116 cells were co-cultured with C2C12 myotubes. Marked myotube atrophy (–53%) was observed, along with elevated phospho-STAT3 levels (+149%). Conversely, inhibition of STAT3 signaling by means of a JAK/STAT3 inhibitor was sufficient to rescue myotube atrophy induced by HCT116 cells (+55%). Overall, our results indicate that the formation of LM exacerbates cachectic phenotype and associated skeletal muscle molecular alterations in HCT116 tumor hosts.

## Abstract (precis)

**Summary:** Colorectal cancer-derived liver metastases exacerbate cachexia, in line with activation of STAT3 signaling. STAT3 inhibition may improve cancer-associated muscle wasting.

## INTRODUCTION

Colorectal cancer (CRC) ranks among the highest for cancer prevalence and mortality within the United States and worldwide (Siegel et al., 2019; Bray et al., 2018). Global deaths attributed to CRC are approaching 1 million annually and, along with the general aging of the population and with the expected increase in CRC incidence in subjects under 50, CRC is expected to become a critical clinical problem (Siegel et al., 2019). In 70% of CRC cases, the most severe complication is the formation of liver metastases (LM), which is often accompanied by the onset of cachexia, a multifactorial syndrome that occurs in up to 55% of all CRC patients (Ruers and Bleichrodt, 2002). Cachexia is defined as an ongoing loss of skeletal muscle mass, with or without the loss of fat mass, which is not fully reversible by conventional nutritional support (Fearon et al., 2011; Siegel et al., 2012; Thoresen et al., 2013). Along with marked muscle wasting, cachexia also induces significant loss of muscle strength, thus contributing to overall functional decline and chemotherapy intolerance in cancer patients (Bruggeman et al., 2016; Fearon et al., 2012). Furthermore, cachexia is directly responsible for 20% of all cancer-related deaths, likely resulting from body weight losses of 25-30%, often progressing into cardiac and respiratory failure (Barton, 2001; Loberg et al., 2007; Tisdale, 2009). Despite its known detrimental impact on patient survival, cachexia research is still underserved and understudied and no treatments are currently available for this debilitating comorbidity of cancer.

A pivotal tool for the study of disease mechanisms and for the identification of novel therapeutic intervention, the use of small animal models has been the preferred choice for cachexia research thus far. However, the delay in developing new and clinically relevant experimental models for the study of cancer cachexia over the past decades has likely also contributed to limited advancement in the field. To date, it is regrettable that only a handful of mouse models are utilized for the study of this syndrome, a majority of which involve the classical Colon-26 (C26) allograft model (Bonetto et al., 2016; Tomasin et al., 2019). Moreover, the current void in the generation of new rodent models of cachexia demands to be filled with new additional models of CRC, in particular metastatic CRC. Ultimately, this approach is also expected to improve translational capacity and to help gain clear insight on mechanisms driving cancer cachexia.

Among the factors that participate in the occurrence of cancer-associated muscle wasting, aberrant pro-inflammatory signaling has received much attention. With respect to CRC, evidence from our laboratory and others has implicated the pro-inflammatory cytokine IL6 as a mediator of muscle wasting in the C26 and Apc^Min/+^ models (Bonetto et al., 2012, 2011; Baltgalvis et al., 2009; White et al., 2011, 2013). In particular, it has been shown that the IL6/STAT3 axis is associated with *in vitro* and *in vivo* muscle wasting, and pharmacologic blockade of STAT3 is sufficient to rescue IL6-induced muscle atrophy (Bonetto et al., 2012; Pin et al., 2018). However, whether STAT3 is also implicated in the occurrence of cachexia in mouse models characterized by the formation of LM CRC needs to be determined.

In the present study we sought to establish new models of CRC cachexia utilizing the HCT116 human CRC tumor line. Here, we demonstrated that subcutaneous or intrasplenic injections of HCT116 cells induce cachexia by promoting differential effects on skeletal muscle. Further, we demonstrate that IL6/STAT3 signaling likely plays a pivotal role in driving muscle wasting by differentially altering pro-anabolic and pro-catabolic pathways in the skeletal muscle of LM HCT116 hosts.

## RESULTS

### HCT116 subcutaneous and metastatic tumor hosts experience weight and fat loss

**Fig. 1.**
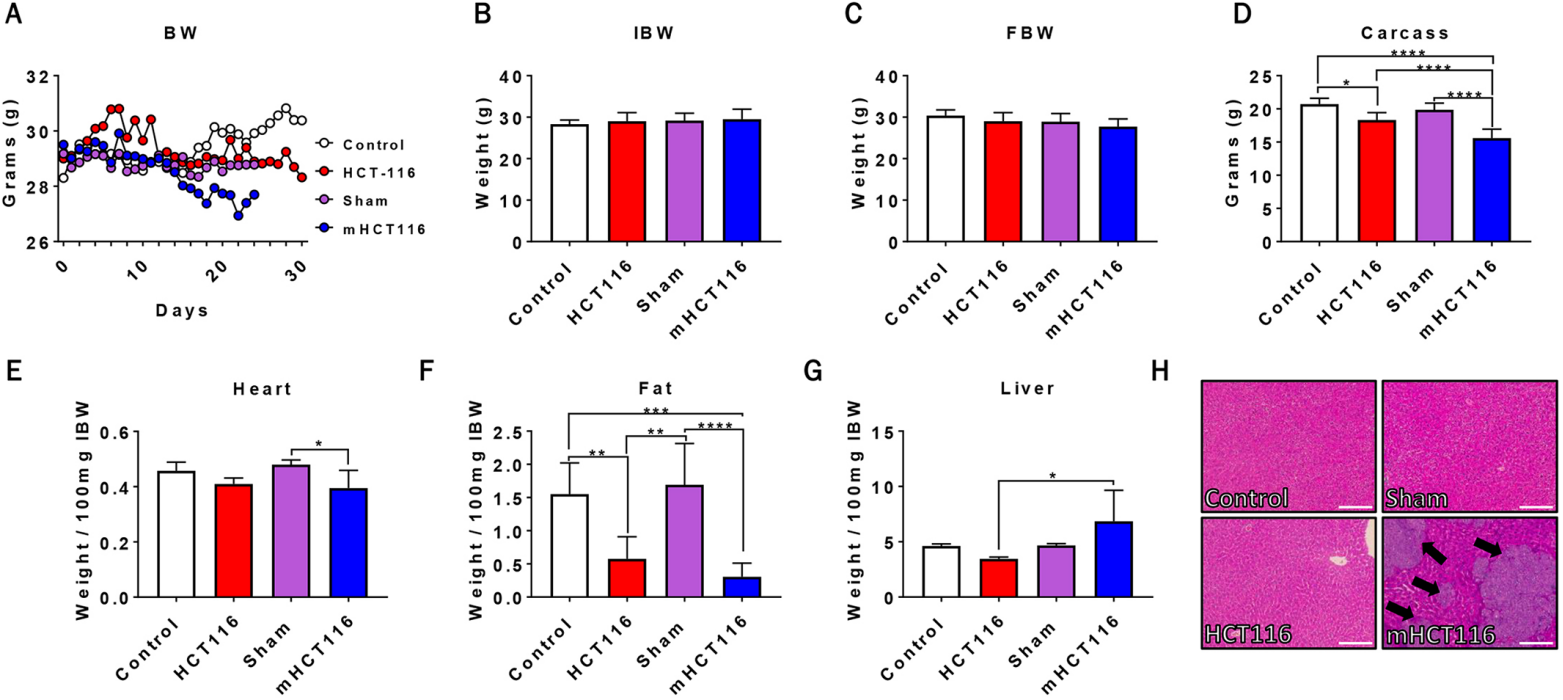
**HCT116 subcutaneous and metastatic tumor hosts experience significant body weight and fat loss.** (A-D) Body weight (BW) curves (A), initial body weight (IBW) (B), final body weight (FBW) (C) and carcass weights (D) of NSG male mice (8-week old) subcutaneously injected with HCT116 tumor cells (3.0×10^6^ cells/mouse in sterile PBS: HCT116) or equal volume of vehicle (control), or intrasplenically injected with HCT116 tumor cells (1.25×10^5^ cells/mouse in sterile PBS: mHCT116) or an equal volume of vehicle (sham) (*n*=5-8). (E-H) Heart (E), fat (F) and liver (G) weights normalized to IBW, and representative H&E staining of liver from control, HCT116, sham and mHCT116 mice (H). Black arrows indicate tumors and images were taken at 10× magnification. Scale bars: 100 µm. Data are mean±s.d. *P<0.05, **P<0.01, ***P<0.001, ****P<0.0001 (one-way ANOVA with Tukey's test).

To assess the impact of HCT116-induced CRC growth on the development of cachexia, male NSG mice were subcutaneously injected with 3×10^6^ HCT116 cells (HCT116) or were intrasplenically injected with 1.25×10^5^ HCT116 cells (mHCT116) to disseminate LM. It is important to note that sham and mHCT116 animals were euthanized at day 24, whereas control and HCT116 animals were euthanized at day 30. By day 24 the mHCT116 hosts were displaying an average weight loss of ∼2 g, which was accompanied by minimal abdominal ascites, marked decline in activity, hunched over appearance, and were therefore euthanized. There was no significant difference in initial or final body weight between experimental groups (Fig. 1A-C). The carcass weights demonstrated a 13% reduction (*P*<0.01, Fig. 1D) in HCT116 hosts relative to controls, whereas they were 21% decreased (*P*<0.0001, Fig. 1D) in the mHCT116 bearers compared with the sham-operated animals. Interestingly, despite being exposed to tumor conditions for a shorter time, the weight of the mHCT116 carcasses was significantly reduced compared with controls (−25%; *P*<0.0001) and HCT116 hosts (−15%; *P*<0.0001). Further, the HCT116 hosts did not display cardiac atrophy, whereas mHCT116 demonstrated significant loss in heart size relative to sham (−18%, *P*<0.05, Fig. 1E). Livers of mHCT116 mice increased 98% compared with livers of HCT116 hosts (*P*<0.05, Fig. 1G), likely owing to the formation of LM (Fig. 1H). In terms of fat mass, mHCT116 hosts saw greater fat loss (−80%, *P*<0.001) relative to controls than did HCT116 hosts (−63%, *P*<0.01) (Fig. 1F), although the two tumor groups were not significantly different.

### HCT116 and mHCT116 hosts display skeletal muscle weakness and atrophy

**Fig. 2.**
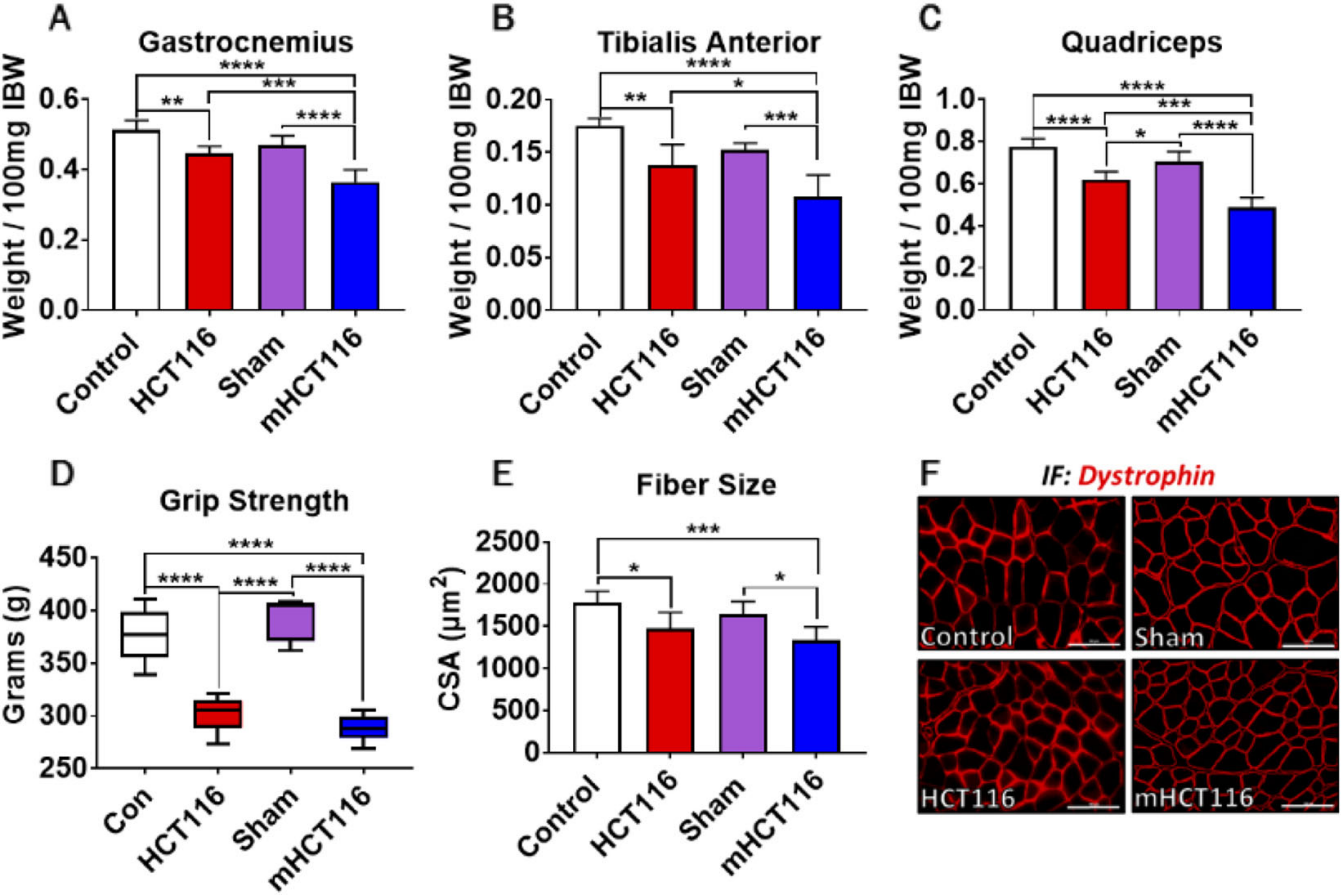
**HCT116 subcutaneous and metastatic tumor hosts experience muscle atrophy and weakness.** (A-C) Gastrocnemius (A), tibialis anterior (B) and quadriceps (C) weights (normalized to initial body weight) of NSG male mice (8-week old) subcutaneously injected with HCT116 tumor cells (3.0×10^6^ cells/mouse in sterile PBS: HCT116) or equal volume of vehicle (control), or intrasplenically injected with HCT116 tumor cells (1.25×10^5^ cells/mouse in sterile PBS: mHCT116) or an equal volume of vehicle (sham) (*n*=5-8). (D-F) Whole-body grip strength assessment (D), cross-sectional area (CSA) of entire tibialis anterior muscles (E) and representative CSA image of tibialis anterior muscle sections stained with anti-dystrophin antibody (F). Scale bars: 100 µm. Data are mean±s.d. *P<0.05, **P<0.01, ***P<0.001, ****P<0.0001 (one-way ANOVA with Tukey's test).

Both HCT116 and mHCT116 tumor hosts revealed marked skeletal muscle atrophy. Compared with controls, the HCT116 hosts displayed significant wasting of gastrocnemius (−13%, *P*<0.05), tibialis anterior (−18%, *P*<0.01) and quadriceps (−20%, *P*<0.001) muscles (Fig. 2A-C). Similarly, mHCT116 hosts also saw significant muscle wasting (gastrocnemius: −23%, *P*<0.0001; tibialis anterior: −29%, *P*<0.001; quadriceps: −31%, *P*<0.0001; Fig. 2A-C) relative to sham animals, although the degree of muscle wasting was more exacerbated in the mHCT116 hosts (gastrocnemius: −19%, *P*<0.001; tibialis anterior: −22%, *P*<0.05; quadriceps: −21%, *P*<0.001 versus HCT116; Fig. 2A-C). Whole-body grip strength assessment demonstrated muscle weakness in both HCT116 (−20%, *P*<0.0001 versus control) and mHCT116 (−27%, *P*<0.0001 versus sham) animals (Fig. 2D). Consistently, the quantification of the cross-sectional area (CSA) in tibialis anterior muscles revealed skeletal muscle atrophy in both tumor hosts (HCT116: −17%, *P*<0.05; mHCT16: −19%, *P*<0.05) compared with the respective control groups (Fig. 2E-F).

### The HCT116 and mHCT116 hosts display altered circulating cytokines

**Fig. 3.**
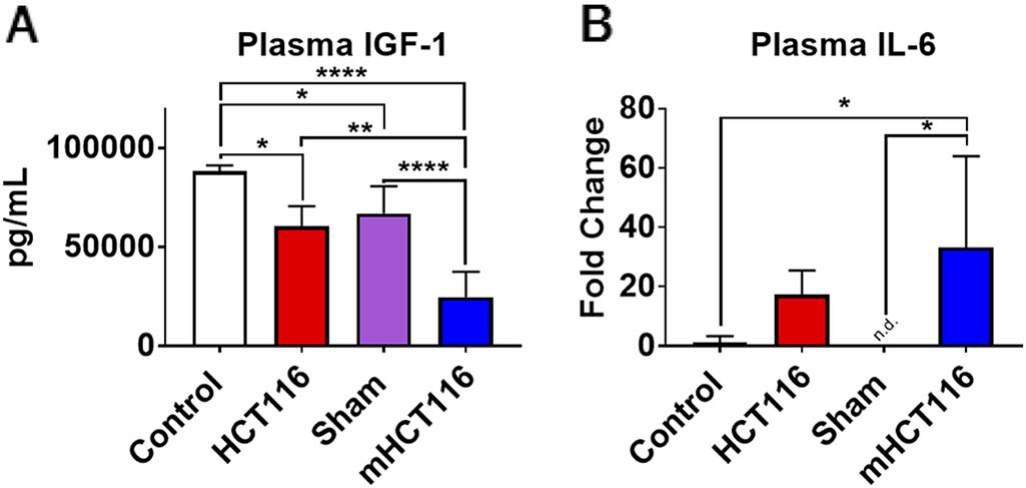
**HCT116 subcutaneous and metastatic tumor hosts experience alterations in circulating cytokines.** (A,B) IGF1 (A) and IL6 (B) levels assessed from plasma of control, HCT116, sham and mHCT116 mice (*n*=5-8) by means of luminex assay. Data are mean±s.d. *P<0.05, **P<0.01, ****P<0.0001 (one-way ANOVA with Tukey's test).

Given that the animals implanted with HCT116 tumors saw a reduction in muscle weight, we sought to assess the circulating levels of known regulators of muscle mass in CRC cachexia, e.g. IGF1 and IL6 (Bonetto et al., 2013, 2012; Costelli et al., 2006; Hetzler et al., 2015; White et al., 2013). We used murine-specific assays to assess the changes in the levels of circulating murine IL6 and IGF1 in the hosts in response to HCT116 growth. Circulating IGF1 levels were reduced by 32% in HCT116 compared with control animals (*P*<0.05). It was also observed that IGF1 levels were reduced in sham animals compared with control (−24%, *P*<0.05). mHCT116 hosts saw marked reductions in circulating IGF1 levels compared with all other groups (−72% versus control, *P*<0.0001; −59% versus HCT116, *P*<0.01; −63% versus sham, *P*<0.0001) (Fig. 3A). On the other hand, only mHCT116 tumor hosts saw significant elevations in circulating IL6 levels with respect to the sham mice (*P*<0.05; Fig. 3B); no changes were detected in the HCT116 hosts.

### mHCT116 hosts demonstrate upregulated skeletal muscle Stat3 phosphorylation

**Fig. 4.**
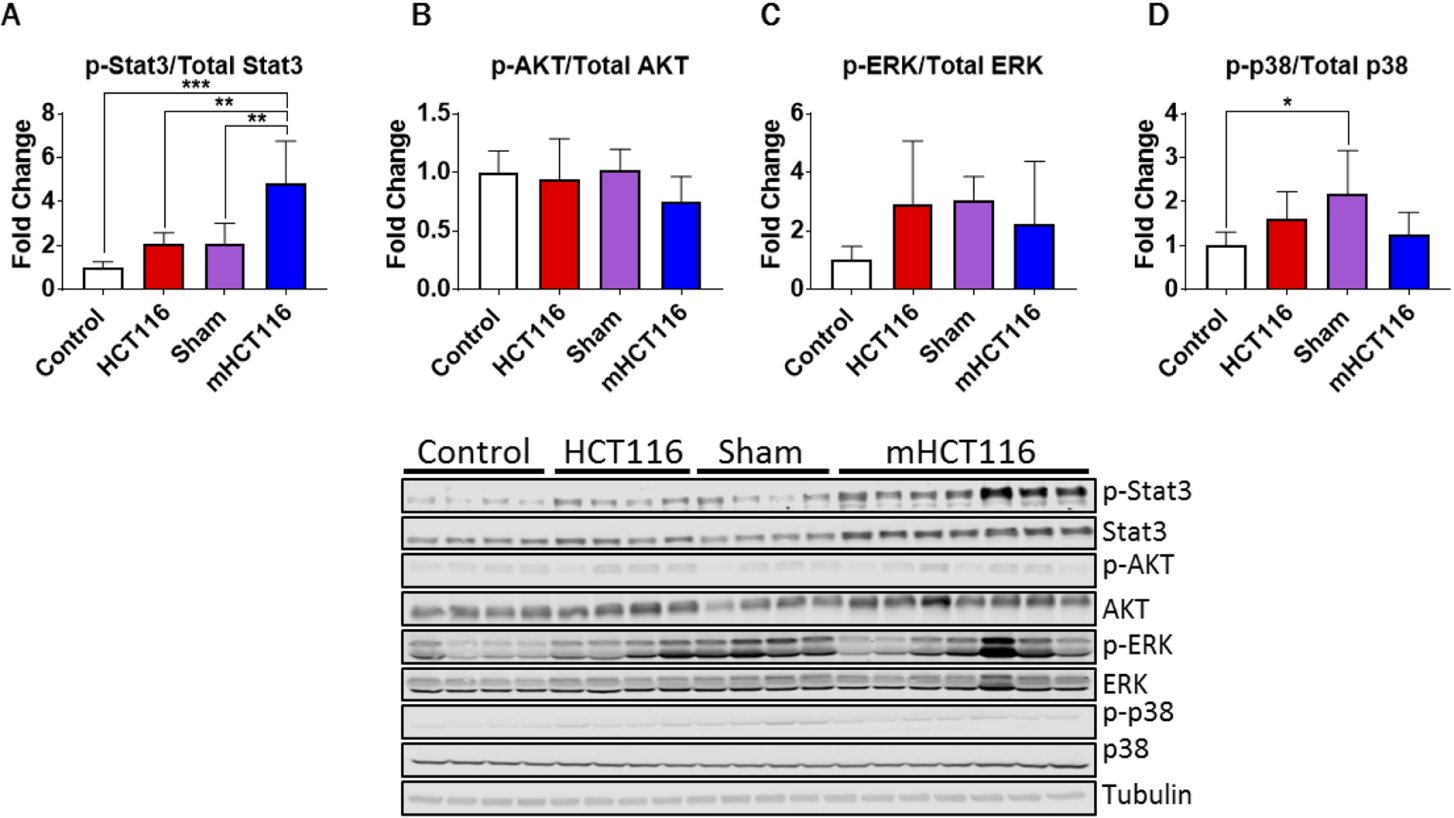
**HCT116 metastatic tumor hosts display elevated STAT3 signaling.** (A-D) Representative western blotting and quantification (expressed as fold change versus control) for phospho-Stat3, Stat3 (A), phospho-AKT, AKT (B), phospho-ERK1/2, ERK1/2 (C), phospho-p38, p38 (D) and tubulin of 8-week-old NSG male mice subcutaneously injected with HCT116 tumor cells (3.0×10^6^ cells/mouse in sterile PBS: HCT116) or equal volume of vehicle (control), or intrasplenically injected with HCT116 tumor cells (1.25×10^5^ cells/mouse in sterile PBS: mHCT116) or an equal volume of vehicle (sham) (*n*=5-8). Data are mean±s.d. *P<0.05, **P<0.01, ***P<0.001 (one-way ANOVA with Tukey's test).

Next, in line with the changes in circulating IGF1 and IL6, we sought to investigate the cachexia-related molecular changes within the skeletal muscle of HCT116 and mHCT116 hosts. In particular, we examined multiple signaling pathways, known to play a critical role in the regulation of muscle size and previously implicated in the pathogenesis of cancer cachexia (Bonetto et al., 2012, 2011; Penna et al., 2010b; Pin et al., 2018). Interestingly, no changes in the phosphorylation of the IGF1-dependent signal transducer AKT (also known as Akt1), as well as of ERK1/2 (Mapk3/Mapk1) and p38 (Ahsa1) were observed in the skeletal muscle of tumor-bearing animals relative to the respective controls (Fig. 4). Contrarily, the sham-operated animals saw an increase in phosphorylated p38 relative to control animals (+117%, *P*<0.05; Fig. 4D), a likely consequence of the surgical procedure. On the other hand, mHCT116 animals experienced drastically increased phosphorylated STAT3 levels compared with all other groups (+384% versus control, *P*<0.001; +132% versus sham, *P*<0.01; +130% versus HCT116, *P*<0.01; Fig. 4A), whereas no significantly increased levels of phospho-STAT3 were detected in the HCT116 hosts compared with control animals.

### mHCT116 hosts exhibit disrupted skeletal muscle mitochondrial homeostasis

**Fig. 5.**
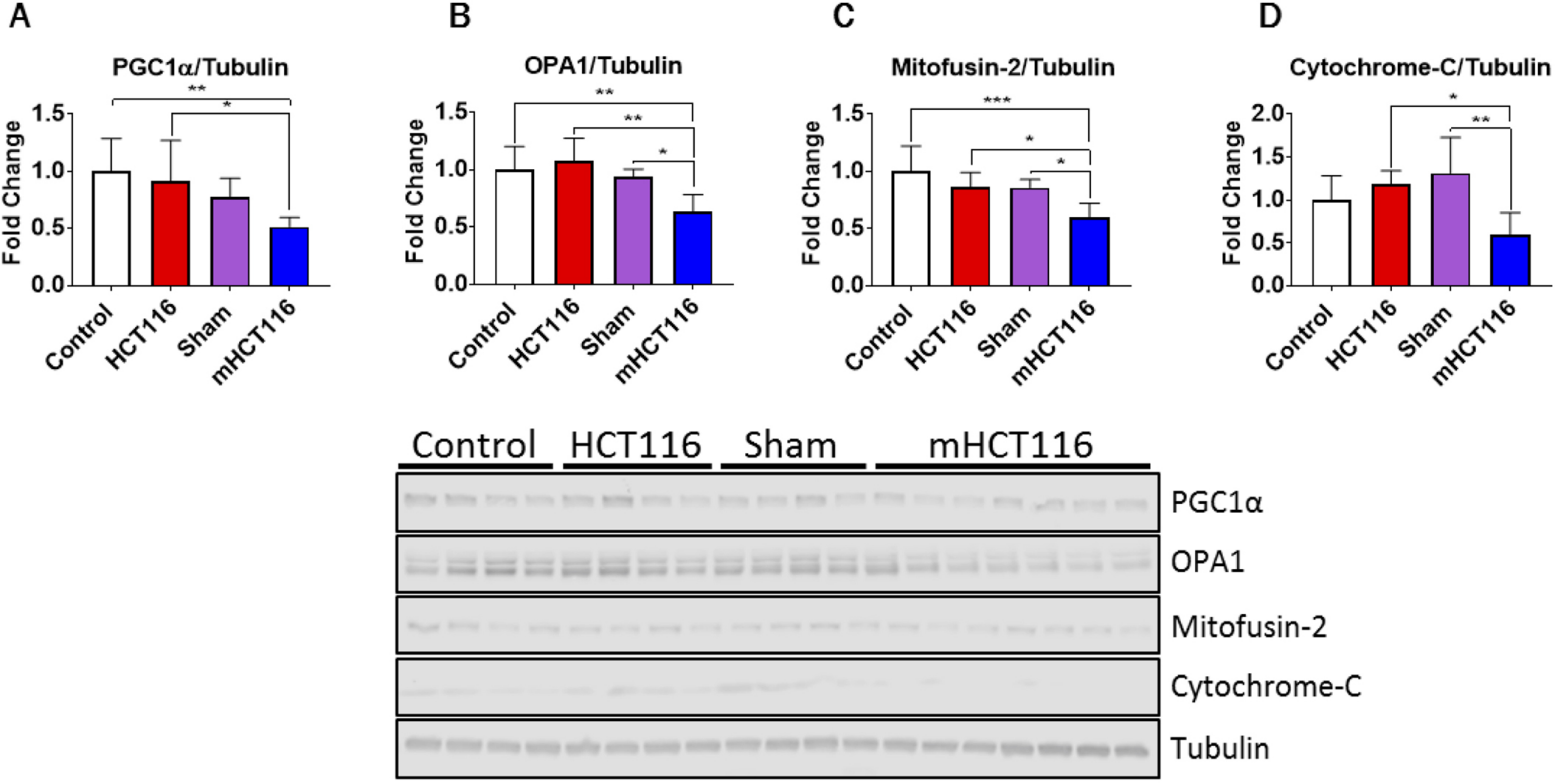
**HCT116 metastatic tumor-bearing mice show mitochondrial disruptions.** (A-D) Representative western blotting and quantification (expressed as fold change versus control) for PGC1α (A), OPA1 (B), mitofusin 2 (C), cytochrome C (D) and tubulin of 8-week-old NSG male mice subcutaneously injected with HCT116 tumor cells (3.0×10^6^ cells/mouse in sterile PBS: HCT116) or equal volume of vehicle (control), or intrasplenically injected with HCT116 tumor cells (1.25×10^5^ cells/mouse in sterile PBS: mHCT116) or an equal volume of vehicle (sham) (*n*=5-8). Data are mean±s.d. *P<0.05, **P<0.01, ***P<0.001 (one-way ANOVA with Tukey's test).

We have demonstrated that cachectic skeletal muscle is often accompanied by perturbations in mitochondrial proteins responsible for mitochondrial fusion and biogenesis (Barreto et al., 2016b; Pin et al., 2018). Here, we sought to examine whether mitochondrial homeostasis was also impaired in animals bearing subcutaneous and liver HCT116 tumors. HCT116 animals displayed no significant alterations in PGC1α (PPARGC1α), OPA1, mitofusin 2 or cytochrome C (Cyc1) compared with control animals (Fig. 5A-D). In contrast, mHCT116 animals displayed significant disruptions in each of these proteins. Indeed, PGC1α was reduced 49% compared with controls (*P*<0.01) and 43% compared with HCT116 hosts (*P*<0.05) (Fig. 5A). mHCT116 hosts showed a 41% reduction of OPA1 compared with HCT116 hosts (*P*<0.01) and 33% compared with sham animals (*P*<0.05) (Fig. 5B). Mitofusin 2 was also reduced in mHCT116 compared with all other groups (−40% versus control, *P*<0.001; −31% versus HCT116, *P*<0.05; −30% versus sham, *P*<0.05) (Fig. 5C). Cytochrome C was reduced in mHCT116 hosts compared with both HCT116 (−50%, *P*<0.05) and sham (−55%, *P*<0.01) (Fig. 5D).

### HCT116 and mHCT116 hosts display elevated skeletal muscle protein catabolism

**Fig. 6.**
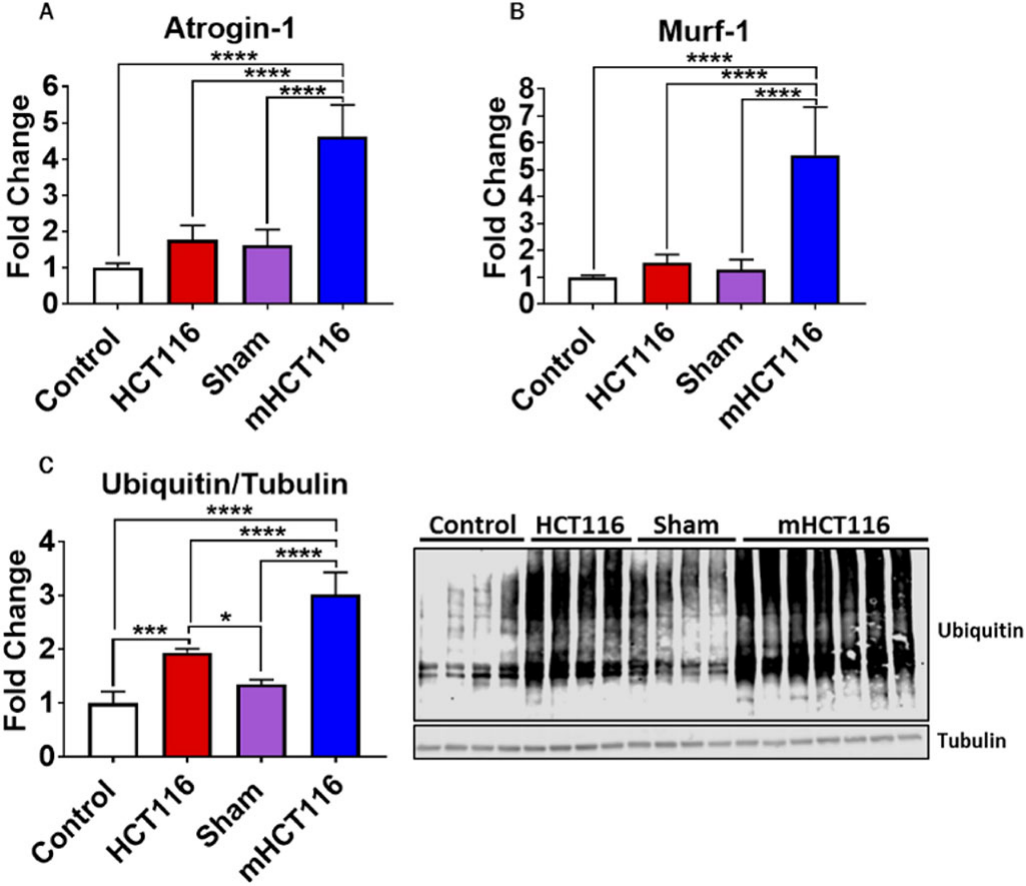
**HCT116 subcutaneous and metastatic tumor hosts exhibit elevated catabolism.** (A-C) Gene expression levels for *atrogin-1* (A) and *Murf1* (B) (normalized to *Tbp*) and representative western blotting and quantification (expressed as fold change versus control) (C) for total ubiquitin and tubulin of 8-week-old NSG male mice subcutaneously injected with HCT116 tumor cells (3.0×10^6^ cells/mouse in sterile PBS: HCT116) or equal volume of vehicle (control) or intrasplenically injected with HCT116 tumor cells (1.25×10^5^ cells/mouse in sterile PBS: mHCT116) or an equal volume of vehicle (sham) (*n*=5-8). Data are mean±s.d. *P<0.05, ***P<0.001, ****P<0.0001 (one-way ANOVA with Tukey's test).

To assess whether muscle wasting in HCT116 and mHCT116 hosts resulted from enhanced protein catabolism, E3 ubiquitin ligases, such as Murf1 (Trim63) and atrogin-1 (Fbxo32) (Bodine et al., 2001), as well as the levels of total ubiquitinated proteins, were assessed. Interestingly, HCT116 mice did not show significant upregulations in the levels of either atrogin-1 or Murf1 (Fig. 6A,B), although they did demonstrate an increase in total ubiquitination compared with control animals (+94%, *P*<0.001) (Fig. 6C). On the other hand, mHCT116 hosts saw significantly increased expression of atrogin-1 compared with all other groups (+359% versus control, *P*<0.0001; +160% versus HCT116, *P*<0.0001; +180% versus sham, *P*<0.0001) (Fig. 6A). Similarly, Murf1 expression exhibited heightened levels in the mHCT116 hosts compared with all other groups (+454% versus control, *P*<0.0001; +259% versus HCT116, *P*<0.0001; +333% versus sham, *P*<0.0001) (Fig. 6B). mHCT116 also demonstrated increased protein ubiquitination compared with sham (+124%, *P*<0.0001) (Fig. 5C).

### IL6/STAT3 plays a pivotal role in HCT116-induced skeletal muscle atrophy

**Fig. 7.**
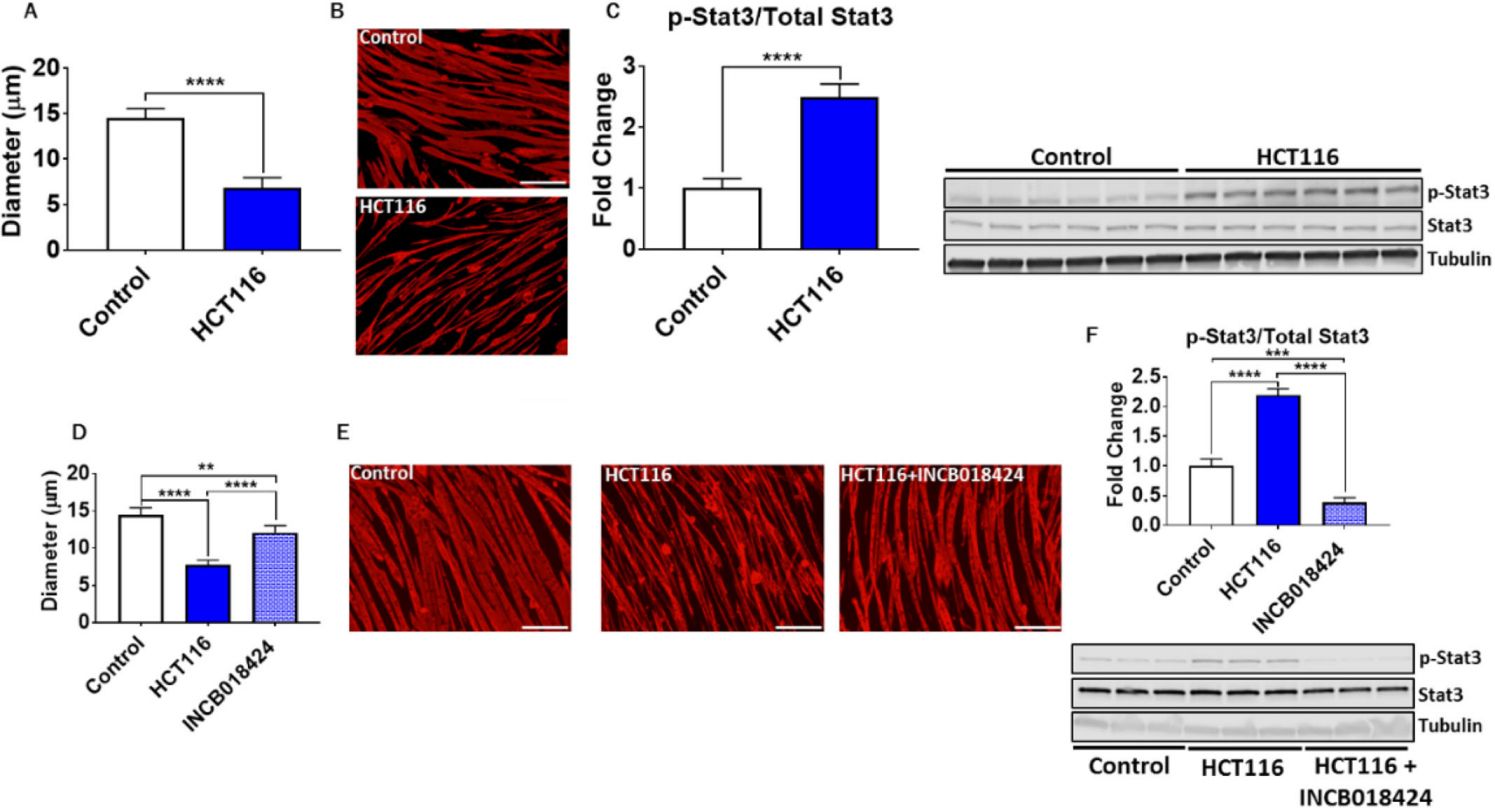
**HCT116 tumor-derived factors induce atrophy of C2C12 myotubes.** (A) Co-culturing of C2C12 myotubes with HCT116 tumor cells for 48 h was performed, followed by assessment of myotube diameter (*n*=350-400). (B) Myotubes were stained with anti-MHC. (C) Representative western blotting and protein quantification for phosphorylated and total STAT3 (*n*=6). (D-F) Follow-up experiments were performed co-culturing HCT116 tumor cells and C2C12 myotubes with or without INCB018424 (400 nM). (D) Quantification of myotube diameter (*n*=350-400). (E) Myotubes were stained with anti-MHC. (F) Representative western blotting and protein quantification for phosphorylated and total STAT3 (*n*=3). Scale bars: 50 µm. Data are mean±s.d. **P<0.01, ***P<0.001, ****P<0.001 (Student's *t*-test: A-C; one-way ANOVA with Tukey's test: D-F).

To determine whether tumor-derived factors may directly contribute to the occurrence of skeletal muscle atrophy, we co-cultured murine C2C12 myotubes with HCT116 cells for 48 h using transwells. Interestingly, co-culturing C2C12 cells with HCT116 tumor cells led to drastic myotube atrophy (−53%, *P*<0.0001) (Fig. 7A,B), accompanied by markedly increased phosphorylation of STAT3 (+149%, *P*<0.0001) (Fig. 7C). In order to determine whether blockade of STAT3 could preserve myotube size in the presence of HCT116 cells, we performed follow-up experiments in which C2C12 and HCT116 cells were co-cultured in the presence or absence of INCB018424, a JAK1/2 inhibitor (Pin et al., 2018). Addition of INCB018424 partially rescued myotube atrophy induced by HCT116 cells (+55% versus HCT116, *P*<0.0001) (Fig. 7D,E), in line with abolished STAT3 phosphorylation (−83% versus HCT116, *P*<0.0001) (Fig. 7F).

## DISCUSSION

CRC is among the deadliest of all cancers in the United States and worldwide (Bray et al., 2018; Siegel et al., 2019) and early detection of CRC is imperative to improve outcomes and overall chances of survival (Levin et al., 2008). Unfortunately, in its most advanced stage CRC is often accompanied by the formation of LM, along with progressive body and muscle weight loss (i.e. cachexia) (Ruers and Bleichrodt, 2002). Notably, the molecular mechanisms responsible for the occurrence of skeletal muscle wasting and its contribution to cancer-related mortality remain partially unknown (Loberg et al., 2007). Among the main reasons for this is the lack of well-characterized and clinically relevant animal models circulating the field, as recently pointed out (Tomasin et al., 2019). This is especially true of CRC-induced cachexia research, in which the C26 subcutaneous model has remained the predominant tool for study over the past decades (Tomasin et al., 2019). Moreover, despite the fact that LM occur in ∼70% of advanced CRC patients, thus also leading to significantly worsened outcomes and poorer survival (Ruers and Bleichrodt, 2002), a limited number of animal models for the study of LM-associated cachexia have been investigated (Tomasin et al., 2019).

In the present study, we utilized the human tumor cell line HCT116 in two separate settings to examine CRC-induced cachexia. In the former, we performed a xenograft transplant of HCT116 cells intrascapularly, similar to the well-characterized murine C26 model (Bonetto et al., 2016), whereas in the latter we injected HCT116 cells intrasplenically to mimic hepatic dissemination of CRC to form LM (Murphy et al., 2013; Ruers and Bleichrodt, 2002). Both subcutaneous HCT116 and mHCT116 tumor hosts demonstrated significant reductions in carcass weight relative to the experimental controls, although we saw greater reductions in skeletal muscle weights in the animals bearing LM, thus demonstrating an exacerbated cachectic phenotype following formation of LM.

The liver, which is crucial in whole-body metabolic homeostasis, has been shown to participate in causing skeletal muscle wasting when compromised (Anand, 2017; Bhanji et al., 2017; De Bandt et al., 2018). Two hepatokines known to play a role in skeletal muscle homeostasis and pathogenesis of multiple diseases, including cancer cachexia, are the anabolic factor IGF1 and the catabolic factor IL6 (Song et al., 2013; Attard-Montalto et al., 1998; Bonetto et al., 2013; Costelli et al., 2006; Cho et al., 2013; Huber et al., 2018). The data presented in the current study support the notion that formation of LM, as occurring in CRC, may further perturb the hepatic production of IGF1 and IL6, thus exacerbating the potential for muscle wasting (Norris et al., 2014; Bonetto et al., 2012, 2011; Ueda et al., 1994; Baltgalvis et al., 2009; White et al., 2013).

In particular, in line with increased circulating IL6 in the mHCT116 tumor hosts, we also reported increased phosphorylated STAT3 levels in the skeletal muscle of mHCT116-bearing animals. Consistently, STAT3 phosphorylation was increased to a greater extent in skeletal muscle of mHCT116 tumor hosts relative to all other groups, including the subcutaneous HCT116 hosts. Of note, tumor hosts did not display changes in the phosphorylation of known regulators of muscle mass in cancer cachexia. As an example, in the present study AKT was unchanged in either context of CRC, despite reduced changes in circulating IGF1. This impairment of the IGF1/AKT signaling pathway was also in line with previously published literature. In support of unchanged AKT, Penna et al. demonstrated no alteration in AKT phosphorylation despite seeing reductions in skeletal muscle IGF1 in the C26 model of CRC cachexia (Penna et al., 2010a). This is in line with our observations, showing reduced circulating IGF1 with unchanged AKT in the skeletal muscle. On the other hand, White et al. demonstrated increased AKT phosphorylation in the Apc^Min/+^ genetic model of CRC (White et al., 2013). In the present study, we also demonstrated no alterations in phosphorylation of ERK and p38 in tumor hosts. ERK phosphorylation has been shown to be differentially changed in several models for the study of cancer- and chemotherapy-induced muscle wasting. For example, we have shown that in ES-2 ovarian cancer ERK phosphorylation is unchanged (Pin et al., 2018), whereas Penna et al. showed increased ERK in the C26 model of CRC (Penna et al., 2010b). Differential findings were also reported relative to p38 regulation in cachexia. Indeed, this signal transducer was increased in the muscle of ES-2 tumor-bearing mice, whereas it was unchanged in the muscle of C26. On the contrary, it was causally linked with the occurrence of muscle atrophy in animals bearing Lewis lung carcinoma (LLC) (Penna et al., 2010b; Pin et al., 2018; Liu et al., 2018). Moreover, we have previously shown that animals administered the commonly used chemotherapeutic regimen Folfiri, routinely prescribed for the treatment of solid tumors, including CRC, present elevated p38 and ERK, consistent with marked muscle wasting (Barreto et al., 2016b).

The fact that these proteins (AKT, ERK, p38) do not change in our study may provide evidence that STAT3 is the main driver of muscle atrophy in the HCT116 model. To validate this concept, we demonstrated that inhibition of STAT3 activation *in vitro* was able to reverse HCT116-induced C2C12 myotube atrophy, in line with our previous observations that inhibition of STAT3 by use of the pharmacologic JAK1/2 inhibitor INCB018424 also rescues myotube atrophy induced by ES-2 ovarian cancer-derived conditioned media (Pin et al., 2018).

STAT3 can serve as a catabolic signal within skeletal muscle (Munoz-Canoves et al., 2013). Here, along with elevated STAT3 signaling, we also observed elevated protein catabolism within the skeletal muscle of mHCT116 hosts, indicated by exacerbated upregulation of the E3 ligases, Murf1 and atrogin-1, and by total protein ubiquitination compared with all groups, all previously shown to be upregulated in cachectic muscle (Kwak et al., 2004; Milan et al., 2015; Pin et al., 2018; Sandri et al., 2004). Interestingly, despite HCT116 hosts displaying muscle wasting and muscle weakness, total ubiquitination was one of the only significantly altered markers in HCT116 hosts, along with reduced serum IGF1. This may indicate that other tumor-derived or host-response factors not measured in this study may be contributing to muscle wasting in HCT116 tumor hosts. On the other hand, we can speculate that the formation of LM might represent the triggering event responsible for significant alterations of the ‘cachexia signature’ in the HCT116 hosts, ultimately leading to a more aggressive cachectic phenotype.

The importance of maintaining mitochondrial homeostasis to sustain muscle mass in disease conditions, such as cancer cachexia, has received much attention (Barreto et al., 2016a; Brown et al., 2017; Pin et al., 2018; Xi et al., 2016). Perhaps of greater interest than the elevation in protein catabolism markers are the differential changes seen in mitochondrial proteins in the two tumor contexts, whereby HCT116 subcutaneous xenografts saw no alteration within the measured mitochondrial proteins and mHCT116 LM hosts saw reductions in PGC1α, OPA1, mitofusin 2, and cytochrome-C. We have recently identified loss of mitochondrial proteins in both cancer and chemo-induced cachexia (Barreto et al., 2016a; Pin et al., 2018). Meanwhile, Brown et al. indicated that mitochondrial dysfunction may precede skeletal muscle loss in LLC, whereas Xi et al. demonstrated that overexpression of mitofusin 2 may be able to partially preserve skeletal muscle in CRC (Brown et al., 2017; Xi et al., 2016). It is plausible that the exacerbated skeletal muscle atrophy in mHCT116 hosts may, at least in part, result from the loss of mitochondrial homeostasis.

Overall, this study clearly demonstrated that formation of HCT116 tumors contributes to the pathogenesis of cachexia in mice, and that LM in CRC exacerbate cachexia, as also supported by the molecular changes consistent with muscle atrophy (e.g. elevated phospho-STAT3, E3 ligases, ubiquitin). In contrast to evidence suggesting that female and male mice display sexual dimorphism with respect to cachexia, we focused our study primarily on male mice, thereby possibly representing a limitation (Hetzler et al., 2015). Moreover, it was demonstrated that female animals in the Apc^Min/+^ CRC model undergo cachexia, at least in part, independently of IL6 (Hetzler et al., 2015). As IL6 is a driver of STAT3 phosphorylation within skeletal muscle, we can only speculate that female mice may not experience the same molecular signatures or cachectic response to formation of LM in this model of CRC (Bonetto et al., 2012; Pin et al., 2018). Hence, future studies will also investigate the muscle phenotype in female animals in contexts of LM associated with CRC-induced cachexia. In addition, other pro-inflammatory cytokines aside from IL6, including IL11 and leukemia inhibitory factor (LIF), are known to activate STAT3-dependent signaling (Heichler et al., 2019; Seto et al., 2015). Such factors were not assessed in the present study, leaving room for future studies to investigate the mechanism by which growth of HCT116 tumors activates STAT3 signaling within skeletal muscle. Moreover, this experimental work narrowly focused on known prognosticators associated with cachexia and, though STAT3 was found to be important in mediating HCT116-induced atrophy, it may not be the sole contributor to muscle wasting in contexts of cancer. Furthermore, though this study demonstrated that LM may exacerbate muscle wasting, additional studies utilizing more comprehensive genomic and proteomic approaches should be performed to better understand how formation of LM may be altering signaling within the skeletal muscle. An additional consideration is how chemotherapy may impact the cachectic phenotype in this model of LM. We and others have previously indicated that chemotherapy alone can induce muscle wasting and weakness (Barreto et al., 2017, 2016a,b; Huot et al., 2019; Hain et al., 2019a,b). As formation of LM exacerbated muscle wasting in the present study, it will be important to examine whether addition of anticancer treatments may further aggravate the loss of muscle mass. Lastly, it is important to note that the animals utilized in this work were immune deficient in order to allow the growth of human tumors. Though this may represent a limitation of the study, implanting human tumors into mice also allows for easy identification between tumor-derived factors and host-response factors.

In conclusion, we have demonstrated that formation of HCT116 subcutaneous and LM xenografts induces muscle wasting in a new animal model for the study of cachexia. However, these data also demonstrate that formation of LM, which are present in many advanced CRC patients, may lead to exacerbation of skeletal muscle atrophy. The exacerbated muscle wasting observed in mHCT116 tumor hosts was accompanied by increased circulating IL6, phosphorylated STAT3, E3 ubiquitin ligases and total ubiquitin, as well as reduced mitochondrial proteins, thereby suggesting that formation of LM in CRC is contributing to muscle wasting via multiple avenues. Moreover, myotube atrophy induced by HCT116 tumor cells was partially rescued by inhibition of STAT3, further suggesting that STAT3 remains an important player in mediating CRC-induced cachexia. Taken together, this study should help pave the way for widespread use of metastatic CRC models to study cachexia, in hopes of better understanding CRC-induced muscle wasting, and ultimately developing therapeutics to improve skeletal muscle mass and survival in CRC patients.

## MATERIALS AND METHODS

### Cell cultures

Human HCT116 cells were purchased from American Type Culture Collection (ATCC) (#CRL-247) and cultured in McCoy's medium supplemented with 10% fetal bovine serum (FBS), 1% glutamine, 1% sodium pyruvate and 1% penicillin/streptomycin (P/S) in 5% CO_2_ at 37°C. Murine C2C12 skeletal muscle myoblasts (ATCC, CRL-1772) were grown in high glucose Dulbecco's modified Eagle's medium (DMEM) supplemented with 10% FBS, 1% P/S, 1% sodium pyruvate and 2 mM L-glutamine while maintained at 37°C in 5% CO_2_. C2C12 myotubes were produced by exposing fully confluent myoblasts to DMEM containing 2% horse serum, 2 mM L-glutamine and 1% P/S, replacing the media every other day for 5 days. To assess the impact of tumor-derived factors on fiber size, HCT116 cells were co-cultured with myotubes using transwell inserts (Thermo Fisher Scientific, #12565009) for 48 h. The JAK1/2 inhibitor INCB018424 (EMD Millipore) was added to culture medium at a final concentration of 400 nM for 48 h in conjunction with the HCT116 co-culture experiments (Pin et al., 2018).

### Animals

Animal experiments were conducted with approval from the Institutional Animal Care and Use Committee at Indiana University School of Medicine (IUSM). Eight-week-old male NOD scid gamma (NSG; NOD-scid/IL2Rg^null^) immunodeficient mice (In Vivo Therapeutics Core Facility, IU Simon Cancer Center, Indianapolis, IN, USA) were housed (up to five per cage) within a pathogen-free facility at IUSM's laboratory animal resource center. Animals were randomized into one of the following experimental conditions: subcutaneous injection of 3.0×10^6^ HCT116 tumor cells in sterile saline (HCT116, *n*=5) or an isovolumetric subcutaneous injection of vehicle (control, *n*=5); intrasplenic injection of 1.25×10^5^ HCT116 tumor cells in sterile saline (mHCT116, *n*=8) or an isovolumetric intrasplenic injection of vehicle (sham, *n*=5). Eight animals were initially enrolled in the mHCT116 group to account for possible complications or early deaths associated with the surgical procedure. However, no unexpected post-operative complications or early deaths occurred and all mHCT116 animals were included in the study. The intrasplenic injections of HCT116 tumor cells and sham-saline injections were performed under aseptic conditions as performed previously (Brand et al., 1996; Xu et al., 2017; Yu et al., 2004). Briefly, mice were placed under anesthesia (2-3% isoflurane) on a heated operating bed, and were unresponsive to touch. Pre-operative slow release buprenorphine (1 mg/kg) was injected subcutaneously in the shank for pain management. A left subcostal incision was made to expose the peritoneum, followed by a small peritoneal incision, exposing the spleen. Then 100 μl of either HCT116 tumor cells or saline was injected to the lateral portion of the spleen using a 26-gauge needle over the period of 1 min. Following injection, the spleen was re-implanted, the peritoneum sutured and the skin closed with surgical staples. Immediately following surgery animals were monitored continuously for 5 h, provided wet feed and then returned to colony. Over the next week animals were checked every 12 h to ensure recovery and staples were removed 7 days post-op. Animals were weighed daily and then euthanized under light isoflurane anesthesia. At the time of euthanasia, skeletal muscle tissues were harvested, weighed and snap frozen in liquid nitrogen and stored at −80°C for further studies. The tibialis anterior muscles were frozen in LN2-cooled isopentane for histology, as previously described (Pin et al., 2018).

### Whole-body grip strength assessment

Whole-body grip strength was assessed using a commercially available automatic grip strength meter (Columbus Instruments) as previously indicated (Bonetto et al., 2015). The absolute force (expressed in g) was recorded over five measurements, with the top three measurements used for analysis. To further avoid habituation bias, animals were only tested once during the experimental period.

### Muscle CSA

To assess skeletal muscle atrophy, 10-μm-thick cryosections of tibialis anterior muscles taken at the mid-belly were processed for immunostaining as described previously (Huot et al., 2019). Briefly, sections were blocked for 1 h at room temperature and incubated overnight at 4°C with a dystrophin primary antibody [1:50, #MANDRA1(7A10), Developmental Studies Hybridoma Bank], followed by a 1 h secondary antibody (AlexaFluor 594, 1:1000, A21125, Thermo Fisher Scientific) incubation at room temperature. Entire dystrophin-stained sections were analyzed for CSA using a Lionheart LX automated microscope (BioTek Instruments).

### Myotube diameter

To assess size, differentiated C2C12 myotubes were fixed in ice-cold acetone:methanol for ten minutes, blocked for 1 h at room temperature and incubated overnight at 4°C with an anti-myosin heavy chain antibody (MF-20, 1:100, Developmental Studies Hybridoma Bank). The following day, myotubes were incubated for 1 h at room temperature with an AlexaFluor 594-labeled secondary antibody (A11032, 1:500; Invitrogen). Myotubes were observed under an Axio Observer.Z1 motorized microscope (Zeiss) and analysis was performed by measuring the diameter of the narrowest portion along the multi-nucleate fibers (*n*=400 fibers per condition) using ImageJ software (Schneider et al., 2012). Three biological replicates (*n*=3) were performed for each experimental condition.

### Western blotting

Skeletal muscle or cell layer protein extracts were obtained by homogenizing 50 mg of quadriceps muscle tissue or entire well surface in ice-cold RIPA buffer [150 mM NaCl, 1.0% NP-40, 0.5% sodium deoxycholate, 0.1% SDS and 50 mM Tris (pH 8.0)] supplemented with inhibitor cocktails for proteases (11697498001, Roche) and phosphatases (78428, Thermo Fisher Scientific). Following a 10-min incubation on ice, cellular debris was removed by centrifugation (15 min, 14,000 ***g*** at 4°C), the supernatant was collected and protein concentration was assessed using the BCA protein assay method (Thermo Fisher Scientific). Protein extracts (30 μg) were electrophoresed in 4-15% gradient SDS Criterion TGX precast gels (Bio-Rad) and transferred to nitrocellulose membranes (30 min at 100 V; Bio-Rad). Following transfer, nitrocellulose membranes were blocked with odyssey blocking buffer (LI-COR Biosciences) at room temperature for 1 h, followed by an overnight incubation with gentle rocking with diluted primary antibody in odyssey blocking buffer containing 0.2% Tween-20 at 4°C. The following day, membranes were serially washed with PBS containing 0.2% Tween-20 (PBST) and the membrane was incubated at room temperature for 1 h with either anti-rabbit IgG (H+L) DyLight 800 or anti-mouse IgG (H+L) DyLight 680 secondary antibodies (1:10,000, 5151S and 5470S, Cell Signaling Technologies). Blots were again serially washed using PBST and then visualized and quantified using the Odyssey Infrared Imaging System (LI-COR Biosciences). Antibodies used were phospho-STAT3 (Tyr705) (1:1000, #9145), STAT3 (1:1000, #12640), phospho-AKT (Ser473) (1:1000, #4060), AKT (1:1000, #9272), phospho-ERK1/2 (Thr202/Tyr204) (1:1000, #4370), ERK1/2 (1:1000, #4695), phospho-p38 (Thr180/Tyr182) (1:1000, #4511), p38 (1:1000, #9212), OPA-1 (1:1000, #80471), mitofusin 2 (1:1000, #9482), cytochrome C (1:1000, #11940) and ubiquitin (1:1000, #3933) from Cell Signaling Technologies; PGC-1α (1:1000, #AB3242) from MilliporeSigma; and α-Tubulin (1:1000, #12G10) from Developmental Studies Hybridoma Bank. In general, phosphorylated protein levels were normalized to the expression of the respective total proteins and tubulin was used as loading control.

### Real-time quantitative polymerase chain reaction (qRT-PCR)

Total RNA from the quadriceps muscle was isolated using the miRNeasy Mini kit (Qiagen) following the manufacturer's instructions. Following extraction, RNA was quantified using a Synergy H1 spectrophotometer (BioTek). RNA integrity was checked by electrophoresis on a 1.2% agarose gel containing 0.02 mol/l morpholinopropanesulfonic acid and 18% formaldehyde. Total RNA was reverse transcribed to cDNA using the Verso cDNA kit (Thermo Fisher Scientific). Transcript levels were measured by Real-Time PCR (Light Cycler 96, Roche) taking advantage of the TaqMan gene expression assay system (Life Technologies). Expression levels for *atrogin-1* (Mm00499523_m1) and *Murf1* (Mm01185221_m1) were detected. Gene expression was normalized to *Tbp* (Mm01277042_m1) levels using the standard 2−ΔΔCT methods.

### Quantification of IL6 and IGF1 in plasma

The circulating levels of mouse IL6 and IGF1 were measured in EDTA-treated mouse platelet-poor plasma via magnetic luminex assay (IL6: LXSAMSM-BR27; IGF1: LXSAMSM-BR55; R&D Systems) according to the manufacturer's protocol.

### Statistics

One-way analysis of variance (ANOVA) tests were performed to determine differences between experimental groups. Post-hoc comparisons were accomplished via a Tukey's test, with statistical significance set *a priori* at *P*≤0.05. When comparing only control and HCT116-treated myotubes, Student's *t*-test was used. All statistics were performed using GraphPad Prism 7.04 and all data are presented as mean±s.d.
